# Dynamics of above- and belowground responses of silver birch saplings and soil gases to soil freezing and waterlogging during dormancy

**DOI:** 10.1093/treephys/tpab002

**Published:** 2021-01-13

**Authors:** Tapani Repo, Timo Domisch, Marja Roitto, Jouni Kilpeläinen, Ai-Fang Wang, Sirpa Piirainen, Juha Heiskanen, Naoki Makita, Tarja Lehto, Sirkka Sutinen

**Affiliations:** Natural Resources, Natural Resources Institute Finland (Luke), Yliopistokatu 6B, PO Box 68, FI-80100 Joensuu, Finland; Natural Resources, Natural Resources Institute Finland (Luke), Yliopistokatu 6B, PO Box 68, FI-80100 Joensuu, Finland; Natural Resources, Natural Resources Institute Finland (Luke), Yliopistokatu 6B, PO Box 68, FI-80100 Joensuu, Finland; Ruralia Institute and Helsinki Institute of Sustainability Science, University of Helsinki, Lönnrotinkatu 7, FI-50100 Mikkeli, Finland; Natural Resources, Natural Resources Institute Finland (Luke), Yliopistokatu 6B, PO Box 68, FI-80100 Joensuu, Finland; College of Horticulture, Hebei Agricultural University, Lekai South 2596, 071001, Baoding City, China; Natural Resources, Natural Resources Institute Finland (Luke), Yliopistokatu 6B, PO Box 68, FI-80100 Joensuu, Finland; Natural Resources, Natural Resources Institute Finland (Luke), Neulaniementie 5, FI-70210 Kuopio, Finland; Faculty of Science, Shinshu University, 3-1-1 Asahi, Matsumoto, Nagano 390-8621 Japan; School of Forest Sciences, University of Eastern Finland, Yliopistokatu 7, FI-80101 Joensuu, Finland; Natural Resources, Natural Resources Institute Finland (Luke), Yliopistokatu 6B, PO Box 68, FI-80100 Joensuu, Finland

**Keywords:** abiotic stress, climate change, greenhouse gas concentration, mortality, phenology, root

## Abstract

Winter precipitation and soil freeze–thaw events have been predicted to increase in boreal regions with climate change. This may expose tree roots to waterlogging (WL) and soil freezing (Fr) more than in the current climate and therefore affect tree growth and survival. Using a whole-tree approach, we studied the responses of silver birch (*Betula pendula* Roth.) saplings, growing in mineral soil, to 6-week Fr and WL in factorial combinations during dormancy, with accompanying changes in soil gas concentrations.

Physiological activation (dark-acclimated chlorophyll fluorescence and chlorophyll content index) and growth of leaves and shoot elongation and stem diameter growth started earlier in Fr than NoFr (soil not frozen). The starch content of leaves was temporarily higher in Fr than NoFr in the latter part of the growing season. Short and long root production and longevity decreased, and mortality increased by soil Fr, while there were no significant effects of WL. Increased fine root damage was followed by increased compensatory root growth. At the beginning of the growing season, stem sap flow increased fastest in Fr + WL, with some delay in both NoWL (without WL) treatments. At the end of the follow-up growing season, the hydraulic conductance and impedance loss factor of roots were higher in Fr than in NoFr, but there were no differences in above- and belowground biomasses. The concentration of soil carbon dioxide increased and methane decreased by soil Fr at the end of dormancy. At the beginning of the growing season, the concentration of nitrous oxide was higher in WL than in NoWL and higher in Fr than in NoFr. In general, soil Fr had more consistent effects on soil greenhouse gas concentrations than WL. To conclude, winter-time WL alone is not as harmful for roots as WL during the growing season.

## Introduction

Winter precipitation has been predicted to increase in northern latitudes in the future (Brown and Mote 2009, IPCC 2018). This will crucially affect boreal forest ecosystems, depending on whether it will take place as snow or rain. Together with more frequent snow melt due to increasing winter temperatures, increased precipitation will decrease insulating snow cover in the areas where it is deep in the current climate (McCabe and Wolock 2010). Consequently, soils may not freeze in regions where there is deep frost in the current climate (Venäläinen et al. 2001, Kreyling 2019). However, low freezing (Fr) temperatures in winter will probably not disappear in northern latitudes (Petoukhov and Semenov 2010). Due to the change in snow cover, the range of soil frost occurrence will probably move to new areas. Soil freeze–thaw events may become more frequent, and soil, water-saturated or not, may freeze more deeply than in the current climate depending on site (Isard and Schaetzl 1998, Groffman et al. 2001, Henry 2008, Kellomäki et al. 2010). Because soil temperature and moisture are the key exogenous drivers of fine root dynamics, it is important to understand the mechanisms by which changes in boreal winter conditions affect soil–plant–climate interactions and the growth and survival of trees accordingly.

Silver birch has a wide distribution, ranging from Mediterranean mountains near to latitude 70° in the north and to central Siberia in the east (Vakkari 2009). It is a pioneer species and typically grows on well-drained soils, as opposed to downy birch, which is more common on poorly drained sites. Due to its wide distribution, silver birch is potentially exposed to a broad range of conditions including soil freezing, the strength and duration of which depends on snow cover and location. If freeze–thaw events become more common in the future, soils even on well-drained sites may be temporarily both water-saturated and frozen. Because the annual cycle of trees is an entity, the environmental conditions and events in one phase of the cycle may affect the development in the subsequent phase(s). Therefore, the stress projected on roots in winter may induce long-term lagged changes in leaf and needle morphology (e.g., surface structure) and growth of above- and belowground organs, but these linkages are not known well (Sutinen et al. 2014, Thitz et al. 2017).

Waterlogging (WL) is harmful for tree roots, because water replaces air in soil, leading to hypoxic or even anoxic conditions (Crawford and Braendle 1996). Survival strategies for WL stress are different during the growing season than during dormancy. Escape mechanisms tend to prevail during the growing season, whereas in winter, survival depends on tolerance by the down-regulation of metabolism (Crawford 2003). Roots are typically less susceptible during dormancy than in the growing season because of low oxygen (O_2_) consumption of cells for energy production in cold soil (Coutts and Nicoll 1990), as found in above zero temperatures for Norway spruce (*Picea abies* (L.) Karst) (Wang et al. 2013) and silver birch (*Betula pendula* Roth.) seedlings in growth chamber experiments (Wang et al. 2016, 2017). In the boreal zone, the most susceptible phase of the roots of Scots pine (*Pinus sylvestris* L.) and Norway spruce seedlings to WL was found to be in the latter part of the growing season, when the roots were still growing (Pelkonen 1975, 1979). In the middle of the growing season, the first symptoms in the roots and shoots of Scots pine saplings were observed after 1–2 weeks of WL but more clearly after 3 weeks of WL (Repo et al. 2016, 2020).

Missing snow cover and the formation of an ice layer in the soil or on the soil surface by freeze–thaw events have consequences for both plant roots and soil microbes (Groffman et al. 2001, 2006, Tierney et al. 2003). Although short-term subzero temperatures may not be harmful for cold-acclimated roots (Bigras et al. 2001, Bigras and Dumais 2005), long-term Fr may worsen the situation (Tierney et al. 2001), especially in water-saturated soil (Sutinen et al. 2014). In snow removal experiments in field conditions, prolonged soil Fr was harmful for several conifer species (Tierney et al. 2001, 2003, Gaul et al. 2008). In a laboratory experiment, root damage by artificial frosts caused a reduction of growth and survival, but roots recovered, depending on the proportion of root damage and species, white spruce (*Picea glauca* (Moench) Voss) being more sensitive to frost than black spruce (*Picea mariana* (Mill.)) and jack pine (*Pinus banksiana* (Lamb.)) (Coursolle et al. 2002). In an experiment using Scots pine saplings with Fr and WL for 6 weeks during dormancy, chlorophyll fluorescence (*F*_v_/*F*_m_) and the water potential of needles were lower, and the apoplastic electrical resistance of needles was higher in Fr than in NoFr (soil not frozen) already during dormancy (Roitto et al. 2019). In the same study, shoot elongation started earlier if exposed to Fr than NoFr conditions, but the onset of fine root growth was delayed by 20 days when Fr was combined with WL. It is not known whether a deciduous species, like silver birch, responds to Fr and WL during dormancy similarly to evergreen Scots pine. Although a missing snow cover and the formation of an ice layer on the soil surface were more harmful for young seedlings of Scots pine than downy birch (*Betula pubescens* Ehrh.) (Domisch et al. 2018, 2019), it is unknown whether the responses of older plants would be similar.

The winter dynamics of greenhouse gases (GHG), i.e., carbon dioxide (CO_2_), nitrous oxide (N_2_O) and methane (CH_4_), are particularly interesting in terms of the ongoing climate change. This is due to a predicted change in soil Fr by altered snow cover and in soil water table by increased precipitation (Groffman et al. 2006). In addition, draining of managed forests to decrease the water table, aiming to increase tree growth, affects GHG fluxes between the soil and atmosphere (Ojanen et al. 2018). Much of the annual N_2_O emission, depending on the ecosystem, has been observed during winter and during the transition from winter to spring, when freeze–thaw events are common (Brumme et al. 1999, Groffman et al. 2000, Maljanen et al. 2007), but the influx of CH_4_ decreased with soil Fr (Groffman et al. 2006). On the other hand, winter fluxes of CO_2_ were less important to the annual flux than those of N_2_O, respectively. This pattern is probably because one primary process of soil CO_2_ production, i.e., root respiration, is likely small during winter (Muhr et al. 2009). Nevertheless, short- and long-term bursts of CO_2_ are caused by different processes during soil thawing in the spring (Kurganova et al. 2007, Maljanen et al. 2010). Greenhouse gases fluxes are linked to the soil water table such that CH_4_ emissions decrease but N_2_O emissions increase with the lowering of the water table (Maljanen et al. 2010, Ojanen et al. 2018). However, the interactive effects of WL and Fr on GHG fluxes between the soil and atmosphere and possible counter effects on physiological processes and the growth of roots and shoots are unknown.

The aim of this study was to obtain an integrated view of the changes in the root zone environment and the mechanisms of the root and shoot responses of silver birch saplings, as affected by WL and soil Fr during dormancy. We hypothesized that although roots are physiologically not very active during dormancy, WL and soil Fr, with accompanying changes in soil gas concentrations, induce lagged changes in the physiology and growth of the roots and aboveground organs during the following growing season.

## Materials and methods

### Plant material and experimental set-up

Sixteen silver birch seedlings (clone L15, origin Loppi, 60°43′N, 24°26′E, southern Finland) were used in the experiment. They were raised in 1-l pots in a greenhouse at the Haapastensyrjä unit of the Natural Resources Institute Finland (Luke) (60°37′N, 24°26′E) for approximately 10 weeks. In the middle of June 2010, they were transported to Luke’s Joensuu unit (62°36′N, 29°45′E, 80 m above sea level) and transplanted in 12-l containers with sieved (10 mm) mineral soil from a birch stand near Joensuu. The seedlings were cultivated in field conditions in Joensuu’s botanical garden for one growing season, when they were fertilized six times at 1-week intervals (lastly on August 2) with summer fertilizer (NPK 13-7-20) (Grow How, Yara Helsinki, Finland) in 0.05% solution with a total nitrogen application of 117 mg per seedling. The seedlings were sprayed twice during the summer against aphids (Cooper Bioruiskute S, 0.2% solution, Berner Ltd, Helsinki, Finland). The seedlings were cold-acclimated outside, where they were chilled for a rest break in buds and attained the competence to commence new growth. At the beginning of December, the seedlings were taken into the Joensuu dasotrons (RTR48, Conviron, Winnipeg, Canada) (Finér et al. 2001) at 4 °C to thaw slowly (including the pots) and replanted in the root containers, four containers in each of four dasotrons. The containers (diameter 70 cm, height 50 cm) were filled with a 15-cm-thick layer of fine textured sand at the bottom and with a 30-cm-thick layer of sieved (10 mm) mineral soil from a birch forest (see above). A 5-cm-thick organic layer from the same stand as the mineral soil was set on the mineral soil. The soil texture in the containers by diameter (*d*) classes was 30% (*d* < 0.063 mm), 22% (0.06 < *d* < 0.2 mm), 26% (0.2 < *d* < 0.6 mm), 12% (0.6 < *d* < 2) and 10% (*d* > 2 mm). The volumetric water content at the matric potentials of −0.1, −1, −10, −100 and −1000 kPa were 46, 40, 26, 13 and 7%, respectively (Heiskanen and Mäkitalo 2002). At the beginning of the experiment, the content of the extractable nutrients, and nitrogen (N), sulfur (S) and phosphorus (P), in the mineral soil on dry mass basis were total-N 0.6 mg g^−1^ and (ammonium nitrogen ((NH4-N) 3.5; aluminium (Al) 409; calcium (Ca) 28; iron (Fe) 22; K 11; magnesium (Mg) 6.5; manganese (Mn) 4.2; sodium (Na) 5.8; P 2.4; S 26) μg g^−1^. Respective values for the humus layer were N 12 mg g^−1^ and (NH_4_-N 51; Al 180; Ca 1320; Fe 62; K 355; Mg 340; Mn 57; Na 14; P 40; S 111) μg g^−1^.

The experimental set-up consisted of two 12-week growing seasons (GS1 and GS2) that included a 3-week short-day period at the end of each season (Table 1). The dormancy period between the growing seasons lasted for 13 weeks, including the transitions between the growing seasons and the dormancy. The first growing season was applied for conditioning of the seedlings to the chamber conditions. In order to compare Fr of waterlogged and not waterlogged soil, the 6-week treatments of WL and Fr were conducted in factorial combinations (Fr + WL, NoFr+WL, Fr + NoWL and NoFr + NoWL, where NoWL stands for ‘without WL’ and NoFr for ‘without Fr’) and took place in the middle of dormancy. The control of soil temperature in the pots took place by stainless steel coils with glycol circulation, one set on the soil surface and another at the bottom of the containers (Finér et al. 2001). In the Fr treatment, the target soil temperature was −2 °C; in the NoFr, it was +2 °C. In the WL treatment, the water table in the containers was raised to the top level of the organic soil by using water from a nearby lake (pH adjusted to 5.5). In the NoWL treatment, as well as during the preconditioning growing season (GS1), the soil water content was maintained near field capacity by irrigation twice a week. The chemical composition of irrigation water was adjusted to correspond to the precipitation in southern Finland (Sallantaus 1992, for ionic composition, see Wang et al. 2013). The Fr treatments were randomly assigned to the chambers, and the WL treatments (subplots) were randomly assigned among the four pots within each chamber. Thus, each chamber had two WL and two NoWL containers. After 6 weeks of WL, the valves at the bottom of the containers were opened for drainage. At the same time, soil thawing started in the Fr treatments. The final harvest took place at the end of the second growing season.

**Table 1:** Air and soil conditions (T refers to temperature) during the experiment’s growing seasons (GS1 and GS2) and dormancy (D) with soil Fr and WL in the middle of dormancy (shaded period). LD is long day, SD is short day, WT is warm temperature, LT is low temperature, RH is relative air humidity, PAR is photosynthetically active radiation, d is day and n is night. Time indicates the starting day of each phase (in running days from the beginning of the experiment). The periods before (PreTrt) and after (PostTrt) the treatments during dormancy lasted for ‘1 + 2’ and ‘3 + 1’ weeks, respectively, both including a 1-week period with a gradual change in air and soil conditions between the growing season and dormancy.

Item	Growing season (GS1)		Dormancy (D)						Growing season (GS2)	
			PreTrt	Fr + WL	Fr + NoWL	NoFr + WL	NoFr + NoWL	PostTrt		
	LD + WT	SD + WT		SD + LT					LD + WT	SD + WT
Starting time, days	0	64	85	106				148	176	239–260
Duration, weeks	9	3	1 + 2	6				3 + 1	9	3
Air T (d/n), °C	20/15	20/15	6/3	4/2				6/3	20/15	20/15
RH (d/n), %	70/80	70/80	90/90	90/90				90/90	70/80	70/80
Photoperiod (d/n), h	18/6	6/18	6/18	6/18				6/18	18/6	18/6
PAR, μmolm^−2^ s^−1^	400	200	200	200				200	400	200
Soil T, °C	15	15	2	--2	--2	2	2	2	15	15

Soil temperature (105 T thermocouple, Campbell Scientific, Shepshed, UK) and volumetric water content (Theta Probe, ML 2×, Delta-T Devices, Cambridge, UK, and CS615, Campbell Scientific) were measured in the mineral soil layer at a depth of 15 cm from the surface at 20-min intervals. The soil O_2_ content in air saturation was measured weekly at the same depth as soil temperature and water content by 4-Channel Fiber-Optic Oxygen Meter (OXY-4, PreSens, Regensburg, Germany), using optical sensors (Oxygen Dipping Probe, DP-PSt3-L2.5-St10-Yop, PreSens). The concentration of CO_2_, CH_4_ and N_2_O in silicon tubes (length 1 m) in equilibrium with soil pores was monitored at the bottom and top of the mineral soil. At sampling, 40–50 ml of air from the tube was sucked into plastic syringes and analyzed on the same day, having been transferred into 6-ml glass vials with a needle (Chromacol®, Sun Sri, Rockwood, TN, USA, caps: BUTYL liner, spring and crimp). Overpressure was avoided by using another needle pushed through the rubber cap of the vial. The gas concentrations were determined using a system of head-space sampler and gas chromatograph (TurboMatrix and Clarus 580 GC, PerkinElmer, Waltham, MA, USA) equipped with a PlotQ capillary column and a two-channel flame ionization detector (FID) that measured CO_2_, CH_4_ and N_2_O.

### Physiological measurements during the experiment

Dark-acclimated (20 min) chlorophyll fluorescence (*F*_v_/*F*_m_) is a measure of the potential photochemical efficiency of photosystem II, whereas the chlorophyll content index (CCI) is a relative value, based on the ratio of transmittances at the 931 and 653 nm wavelengths. The measurement of *F*_v_/*F*_m_ (MINI-PAM, Heinz Walz Gmbh, Effeltrich, Germany) and CCI (CCM-200 plus Chlorophyll Content Meter, Opti-Sciences, Inc., Hudson, NH, USA) took place in one intact leaf from both short and long shoots in the upper and lower part of the canopy at 1-week intervals during the second growing season. The measurements started when the newly flushed leaves were large enough for the measurements.

Gas exchange was measured at light saturation in one leaf in the upper and lower parts of the canopy at 1-week intervals during the second growing season (ADC-LCpro+, Portable Photosynthesis System, ADC Bioscientific Ltd, Hoddesdon, UK). The conditions in the leaf chamber (area 6.25 cm^2^) were 20.0 °C for leaf temperature, 800 μmol m^−2^ s^−1^ for photosynthetically active radiation, 380 μmol mol^−1^ for CO_2_ concentration and 1.6 kPa for water vapor pressure. For each leaf, 10 readings were recorded, and their means were used to calculate the light-saturated net assimilation rate (*A*_max_), transpiration rate (*E*), stomatal conductance (*g*_s_) and water-use efficiency as WUE = *A*_max_/*E*.

The starch content of leaves was assessed at 2-week intervals during the second growing season for two leaves of both short and long shoots. The leaves were packed in aluminum foil, frozen in liquid nitrogen and stored at −80 °C. Before the analyses, the leaves were milled to a powder in liquid N_2_. The starch was extracted from the residue, using 30% perchloric acid, and the dry mass content was determined spectrophotometrically at 625 nm, using anthrone and starch (starch soluble—pro analysis, Merck, Darmstadt, Germany) in 30% perchloric acid as a standard (Hansen and Møller 1975).

Stem sap flow was monitored using a Dynamax Flow32^tm^ Stem-Flow Gauge system (Dynamax Inc., Houston, TX, USA). The Dynamax gauges (SGB9-WS) were installed approximately 10 cm above the root collar. Before the sensor was installed on the stem, the surface was sprayed with a Teflon liquid (Ease-Release Teflon, model no. TFE), and a thin layer of silicon grease was spread on the inner surface of the heater element (van Bavel et al. 2000). The power input was approximately 0.1 W. The thermal conductance of the stem was calculated as the mean value within 2 h of switching the lights on in the morning (van Bavel et al. 2000). The threshold for temperature difference between the upper and lower thermocouple in the gauge was set to 0.8 °C. Below this value, the sap flow rate was assumed to be zero. Data logging took place at 15-min intervals to calculate the daily sap flow. Possible gaps in the 15-min time series were filled by linear interpolation (gaps ≤1 h) and mean diurnal variability methods (e.g., Reichstein et al. 2005). The sap flow data in the second growing season were analyzed by phases. Phase P1 (Days 176–200) is the beginning of the growing season with the leaf unfolding, P2 (Days 201–220) is the leaf expansion phase, P3 (Days 221–237) is the leaf maturity phase and P4 (Days 238–250) is the short-day phase at the end of the growing season.

### Growth measurements during the experiment

The elongation of the leader shoot during the second growing season was measured with a ruler at 1-week intervals. The stem diameter was measured at 8 cm above the root collar with a slide gauge at 1-week intervals. Both the shoot elongation and stem diameter were presented as the percentage of change from the length and diameter at the beginning of the second growing season, respectively.

The leaf expansion during the second growing season was monitored by imaging two leaves of each sapling, the same leaf in the upper and in the lower part of the canopy at 1-week intervals. The leaf area was measured as the number of pixels (*n*_1_), transformed into metric units (*A*_leaf_ in unit mm^2^), on the basis of the number of pixels in the calibration area (100 mm^2^) (*n*_2_) on the coordinate paper by means of the equation *A*_leaf_ = (*n*_1_/*n*_2_) × 100 (Adobe Photoshop CS6, Adobe Systems Nordic AB, Kista, Sweden).

Root growth was followed by means of minirhizotron imaging (Bartz BTC-100X Camera System, Bartz Technology Company, Santa Barbara, CA, USA)—twice during the first growing season, four times during the dormancy and five times during the second growing season. The imaging tube (acrylic glass, exterior diameter 60 mm) was installed horizontally, coincident with filling of the containers and planting, in the mineral layer at a depth of 15 cm from the soil surface. Digital images of the roots were taken in an upward direction along the entire extension of the tube, with a total of 46 frames (13 mm × 18 mm). Using the RootView software (Aphalo and Simonic 1999), fine root appearance, elongation and disappearance/death were assessed separately for the short (first order) and long (higher than first order) roots. A root was defined as dead when it started to appear disintegrated in the image and then disappeared. If the first order root changed to a higher order in any phase during the study, its order was changed retrospectively for all imaging times. For the analysis of the minirhizotron data, the length of roots in all frames of each root container, i.e., each tube (one tube per container), was aggregated by imaging time. The variation between the root lengths (i.e., the data vectors $x$) of each root container was considered by equalization, using the Euclidian norm, whereupon it was possible to observe small changes in the production.

(1) \begin{eqnarray*} X &=& \left\{\frac{x_1}{\sqrt{x_1^2+{x}_2^2+\dots +{x}_n^2}},\frac{x_2}{\sqrt{x_1^2+{x}_2^2+\dots +{x}_n^2}},\dots,\right. \nonumber \\ &&\left. \frac{x_n}{\sqrt{x_1^2+{x}_2^2+\dots +{x}_n^2}}\right\}, \end{eqnarray*}

where $X$ is the normalized root length at the imaging times *t =* {*t*_1_, *t*_2_, …*, t_n_*}. At each time, the production of short and long roots was calculated as the difference between the subsequent normalized lengths.

The proportion of dead fine roots out of the total roots present, termed as mortality, was assessed separately for short and long roots for each imaging time throughout the experiment. The survival probability of short and long roots was analyzed, using Kaplan–Meier statistics (Fay and Shaw 2010).

### Measurements of leaves and roots at the end of the experiment

**Figure 1.**
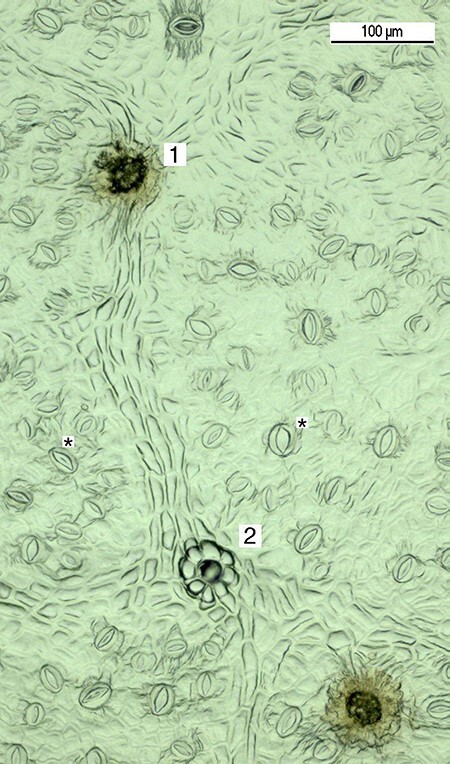
Microscopic images of surface structure of birch leaf, indicating glandular (1) and non-glandular (2) trichomes and stomata (^*^). The scale is indicated by the bar in the upper right-hand corner.

The glandular and non-glandular trichomes (hairs) on the upper and lower sides of two leaves of short and long shoots of each sapling were analyzed at the end of the long-day phase of the second growing season. The number of stomata on the lower side of the short shoot leaves was analyzed at three sampling times, i.e., at the beginning of the second growing season, before the start of the short-day period and at the end of the short-day period, and only at the second sampling time for long shoot leaves. The area from the leaf edge up to the central vein between the two side veins in the middle of each leaf was copied with clear nail polish onto transparent tape from both sides of the leaf, placed on the objective glass, and photographed in three sections, i.e., near central vein, near leaf edge and between them (Leica Microsystems CD Camera, Heerbrugg, Switzerland) under a light microscope (Leica DM2500, Wetzlar, Germany, 20× magnification for the stomata and 2.5× magnification for the glandular and non-glandular trichomes) (Figure 1). Glandular trichomes were characterized by lighter outer cells and a darker base, as described previously (Valkama et al. 2004), and non-glandular trichomes by hairs pointing outward from the marguerite-like base structure, as described by Wang et al. (2016). The number of glandular and non-glandular trichomes was counted for the 3.88 mm^2^ area and the number of stomata for the 0.24 mm^2^ area of each three photographed sections (Adobe Photoshop CS6, Adobe Systems Nordic AB).

Reverse-flow root hydraulic conductance (expressed in mg MPa^−1^ s^−1^) was measured at the final harvest, using a High Pressure Flow Meter (HPFM, Dynamax, Inc.). The stem was cut at about 50 mm above the root collar. The bark was peeled off for 30 mm below the cut point of the stem, and the capillary tube of the HPFM was connected to the cut surface with a coupling set. Air was removed from the cut surface and the space in the coupling by injecting de-aerated water into the coupling, before connecting it with the capillary tube of the HPFM. The measurement was based on monitoring the flow of water by gradually increasing pressure (ranging from 0 to 0.5 MPa) (Tyree et al. 1995). The reverse-flow hydraulic conductance was obtained from the linear part of the relation between water flux and applied pressure.

The effects of the treatments on the electrolyte balance of roots were studied by electrical impedance spectroscopy. The real (*Z*_Re_) and imaginary (*Z*_Im_) parts of the impedance spectra of the root system, the stem above the root collar and soil were measured at the final harvest. The measurement took place at 42 frequencies between 80 Hz and 1 MHz with the circuit analyzer (HP 4284 A, Agilent, Palo Alto, CA, USA) by two-electrode configuration. One paired electrode (two stainless steel needles, diameter 1.5 mm) was set on the opposite sides of the stem at the root collar. In the measurements of the root system, another electrode (stainless steel rod, diameter 4 mm) was set in the soil 30 cm from the stem (Repo et al. 2014*a*, 2016). The measurements were repeated twice, with the stem electrodes switched to a perpendicular position between the measurements. In measuring the stem, another electrode was set in the stem 15 mm above the electrode at the root collar. The measurement of the soil took place between stainless steel electrode rods in the soil. The impedance loss factor ($\delta$) was calculated at 50 kHz frequency (β-dispersion range; for dispersion ranges, see Schwan 1988) (Di et al. 2019) as:

(2) \begin{equation*} \delta ={\tan}^{-1}\left(\frac{Z_{\mathrm{Im}}}{Z_{\mathrm{Re}}}\right) \end{equation*}

To assess root biomass and morphology, a 385 cm^2^ soil sector (10% of the total area) of the top organic layer (at a depth of 5 cm) and three 10-cm-thick mineral soil layers to a depth of 35 cm was harvested from each container at the final harvest. The roots were separated from the soil, dried at 40 °C and weighed. The biomass of the whole root system in the 35-cm layer was extrapolated by considering the volume of the sector sample. The rest of the root system, including the fine and coarse roots and stump, was washed with water and assessed for dry mass as above. The biomass of those roots was added to the extrapolated root biomass to calculate the total root biomass (DM_Tot_). The biomass of leaves and branches was measured at the final harvest after drying at 60 °C.

### Statistical analyses

The effects of the WL and Fr treatments, time and their interactions were analyzed by means of a linear mixed model (procedure MIXED in IBM SPSS Statistics 22.0, IBM Co., Armonk, NY, USA) as:

(3) \begin{eqnarray*} y\!\!\! &=&\!\!\!\! \mu +\mathrm{wl}+\mathrm{temp}+\mathrm{Time}+\mathrm{wl}\times \mathrm{temp}+\mathrm{wl}\times \mathrm{Time}+\mathrm{temp} \nonumber \\ &&\!\!\!\!\!\!\!\times \mathrm{Time}+\mathrm{temp}\times \mathrm{wl}\times \mathrm{Time}+\mathrm{chamber}+\mathrm{sapling}+\varepsilon \end{eqnarray*}

where μ is a constant, wl (WL, NoWL) and temp (Fr, NoFr) are fixed factors, Time is a repeated factor and chamber, sapling and residual Ɛ are random terms. The time correlation of the residuals between the sampling times was described by a heterogeneous ARH1 covariance structure. The difference between the treatments at different sampling times was compared, using Bonferroni-corrected pairwise tests. The distribution of the residuals was checked graphically, and the values were ln- or arctan-transformed when residuals were unevenly distributed. The time correlation structure was selected based on Akaike’s information criteria. The data collected at the final harvest were analyzed by two-way analysis of variance (IBM SPSS Statistics, GLM Univariate) with factorial combinations of the fixed factors Fr and WL. The leaf sides for trichomes were tested with a paired samples *t*-test within each treatment combination.

Root longevity and survival analyses were conducted for short roots and long roots separately by generalized Kaplan–Meier statistics, using the ‘interval’ package in R (version 3.6.1). The appearance and death of individual roots were observed at 3-week intervals, which resulted in an interval, as well as right-censored data. The plots were plotted, using the ‘icfit’ function, and the differences between the treatments were tested with the ‘ictest’ function, using an asymptotic logrank *k*-sample test (permutation form and Sun’s scores, Fay and Shaw 2010). The Kaplan–Meier estimator is typically ‘undefined’ after the last observation if that observation is right-censored (Fay and Shaw 2010), as in our case, for all roots that were alive at the end of the experiment. According to Fay and Shaw (2010), this is because the nonparametric maximum likelihood estimation (NPMLE) is not unique in this case, as changes in the survival distribution after that last censored observation do not affect the likelihood of the observed data. In interval-censored data, the estimate is undefined due to non-uniqueness at certain intervals, and the survival curves plotted with the interval package are shown as descending slopes in these cases (Fay and Shaw 2010). Survival probability plots were calculated using the Survfit-function of the ‘survival’ package to assess the median root longevities when appropriate.

## Results

### Soil and air conditions

**Figure 2.**
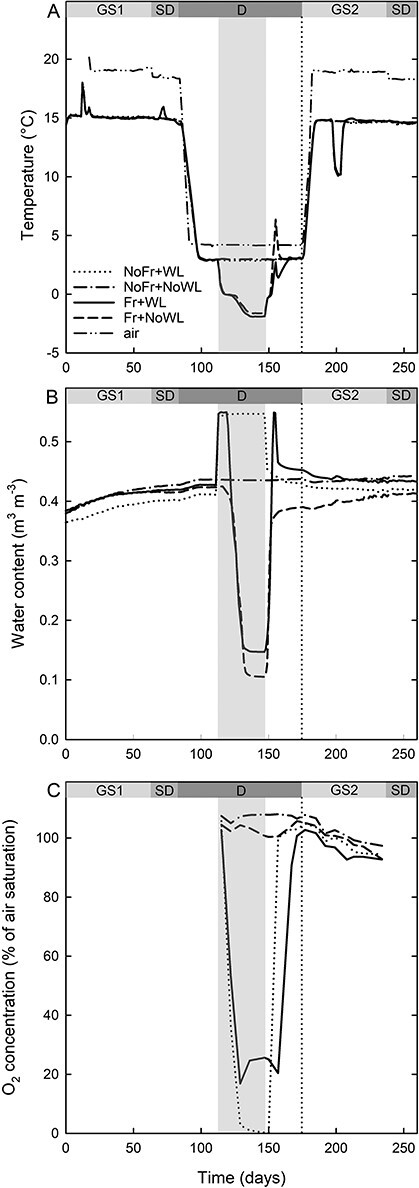
The daily mean temperature of air and soil (A), and the volumetric moisture content (B) and oxygen content (C) of the soil (at a depth of 15 cm from the soil surface) in the experiment, with silver birch in laboratory conditions with factorial combinations of WL and soil Fr during dormancy (D). GS1 and GS2 refer to the growing seasons, including the short-day (SD) periods. The vertical dotted line indicates the start of GS2. Time indicates running day from the beginning of the experiment.

The daily mean air temperature was 18 °C during the growing season and 4.5 °C during dormancy (Figure 2A). The soil temperature was 15 °C during the growing season and a minimum of 2 °C and −2 °C in NoFr and Fr during dormancy, respectively. The soil moisture content changed with some delay after soil Fr and thawing (Figure 2B). The soil oxygen content in the mineral layer responded rapidly to the onset and cessation of WL, with some delayed responses in Fr + WL after soil thawing (Figure 2C).

**Figure 3.**
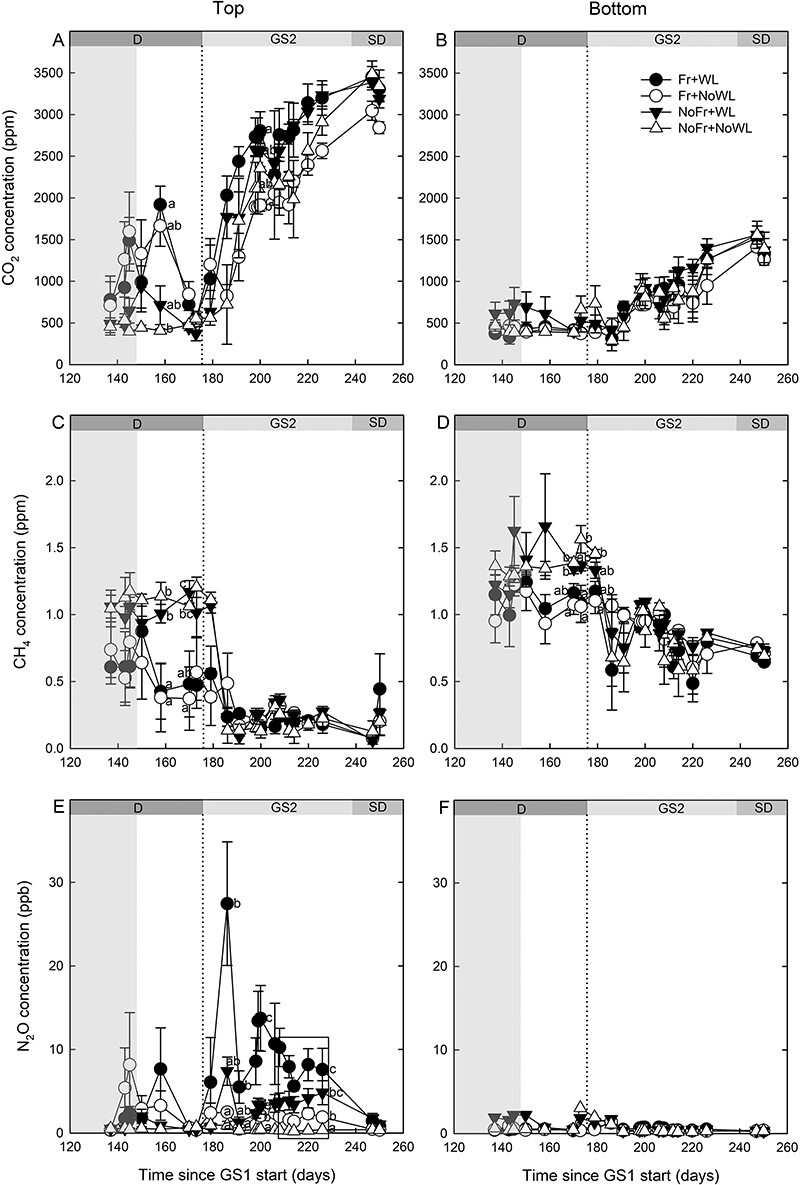
The concentration of CO_2_ (A, B), CH_4_ (C, D) and N_2_O (E, F) in the soil at the top and bottom layers of the containers by treatments, respectively, in the experiment with silver birch saplings, with factorial combinations of soil Fr and WL during dormancy (D). GS2 refers to the growing season including the short-day (SD) period. The latter part of the treatment period is indicated by the shaded column. Significant differences by sampling times are indicated by different letters (*P* < 0.05). The differences between the treatments within the box in (E) are the same. The vertical dotted line indicates the start of GS2. Time indicates running day from the beginning of the experiment. Bars indicate standard errors (*N* = 4).

Waterlogging increased the soil CO_2_ concentration compared with NoWL during the second growing season at the top but not at the bottom of the container (Table 2, Figure 3A and B). The interaction of soil Fr with time had a significant effect on soil CO_2_ at the top layer, with CO_2_ concentration temporarily higher in Fr than in NoFr at the end of dormancy. Waterlogging had no effect on CH_4_ concentration, but it was lower in Fr than in NoFr during dormancy (Figure 3C and D). Those differences disappeared when the growing season started. In both layers, temporarily at the time when the growing season had started, soil N_2_O was higher in WL than in NoWL and higher in Fr than in NoFr (Table 2, Figure 3E and F). There were no interactions between the two treatments except for CH_4_ in the top layer (Table 2).

**Table 2:** The significant (*P* ≤ 0.05 in bold) or close to significant (*P* ≤ 0.1) effects of soil Fr and WL during dormancy on silver birch saplings during the follow-up growing season (GS2) as assessed by linear mixed model. The density of non-glandular and glandular trichomes was analyzed separately for upper (‘up’) and lower (‘down’) surfaces of the leaves of short and long shoots. ‘Short’ and ‘long’ roots refer to the first and higher than the first order roots, respectively. The full table for all variables with degrees of freedom and *F*-values are given in Table S2 available as Supplementary data at *Tree Physiology* Online. Symbols and abbreviations are explained in the text.

	Fr	WL	Fr × WL	Fr × Time	WL × Time
Shoot physiology and growth					
*F*_v_/*F*_m_ (short)	**0.046**	0.89	0.20	**<0.001**	0.08
*F*_v_/*F*_m_ (long)	0.70	0.38	0.27	0.43	**0.02**
CCI	**<0.001**	0.22	0.35	**<0.001**	**<0.01**
Starch (short)	0.10	0.44	0.62	**0.04**	0.58
Sap flow (Phase 1)	0.54	0.06	0.66	**<0.001**	**<0.001**
Sap flow (Phase 2)	0.90	0.17	0.16	**<0.001**	0.62
Shoot elongation	0.20	0.40	**0.02**	**<0.001**	0.08
Stem diameter growth	**<0.001**	**0.03**	0.77	**<0.001**	0.13
Leaf expansion	**0.003**	0.38	0.10	**<0.001**	**0.046**
DM (stem and branch)	0.061	0.786	0.175	n.d.	n.d.
Leaf morphology					
Non-glandular (short, up)	**0.004**	0.46	0.57	n.d.	n.d.
Non-glandular (long, up)	0.07	0.08	0.43	n.d.	n.d.
Glandular (short, down)	0.10	0.64	0.33	n.d.	n.d.
No. of stomata (short)	0.94	0.63	0.06	0.57	0.71
Roots					
Production (short)	0.91	0.53	0.055	**0.001**	0.08
Production (long)	0.52	0.15	0.89	**0.005**	**0.04**
Mortality (short)	0.54	0.83	0.26	**<0.001**	0.55
Mortality (long)	0.43	0.76	0.66	**<0.001**	0.32
Loss factor (EIS)	**0.002**	0.33	0.82	n.d.	n.d.
Hydraulic conductance	**0.001**	0.08	0.80	n.d.	n.d.
DM (root)	0.057	0.460	0.663	n.d.	n.d.
Soil gases					
CO_2_ (top)	0.41	**0.008**	0.54	**0.01**	**<0.001**
CO_2_ (bottom)	0.09	0.15	0.69	0.054	0.38
CH_4_ (top)	**0.028**	1.0	**<0.001**	**0.001**	0.59
CH_4_ (bottom)	**0.018**	0.72	0.20	**0.007**	0.056
N_2_O (top)	0.052	**<0.001**	0.35	**<0.001**	**<0.001**
N_2_O (bottom)	0.59	**0.02**	0.32	**0.012**	**0.024**

### Physiological measurements of leaves and stems

**Figure 4.**
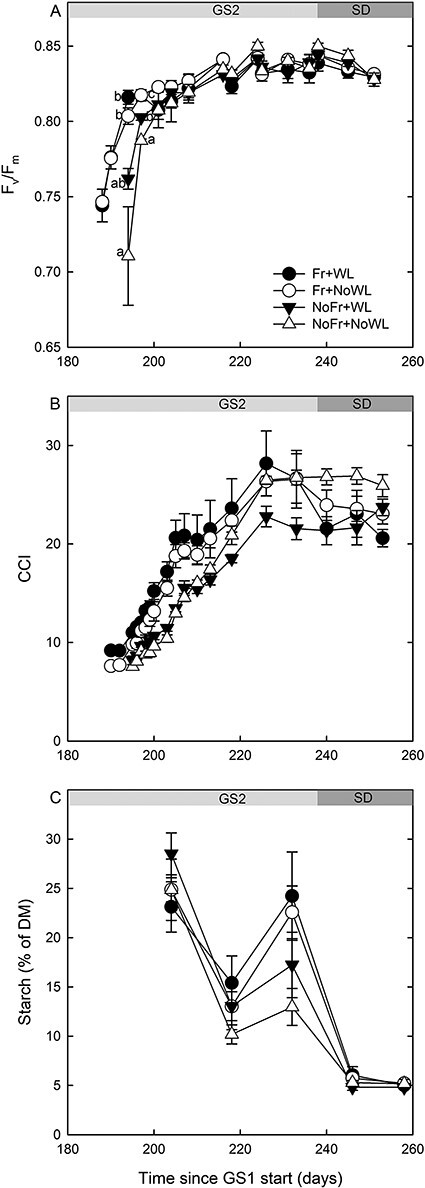
Dark-acclimated chlorophyll fluorescence (*F*_v_/*F*_m_) (A), CCI (B) and starch content (C) of short shoot leaves of silver birch saplings during growing season GS2, including the short-day period (SD), in the experiment, with factorial combinations of WL and soil Fr during dormancy (see Table 1). For clearance, the significant differences by sampling times are indicated by different letters in (A) only (*P* < 0.05) (for the (B) and (C), see the text). Time indicates running day from the beginning of the experiment. Bars indicate standard errors (*N* = 4).

The effects of soil Fr and its interaction with time were significant for the dark-acclimated chlorophyll fluorescence (*F*_v_/*F*_m_) of the leaves of short shoots (Figure 4A, Table 2). A pairwise comparison revealed that *F*_v_/*F*_m_ was higher in the treatment with soil Fr than without Fr at several sampling times at the beginning of the second growing season. In that period, *F*_v_/*F*_m_ was also temporarily higher with than without WL. In long shoots, soil Fr had no effect on *F*_v_/*F*_m_, but the interaction of WL with time was significant (Table 2), with *F*_v_/*F*_m_ being lower in WL than NoWL treatment at the beginning of the short-day period of the second growing season.

According to the pooled data of the leaves in the upper and lower position, soil Fr affected the CCI, but WL did not (Figure 4B, Table 2). The interactions of Time × Fr and Time × WL were significant, indicating that the CCI was higher with than without soil Fr for 3 weeks and higher with than without WL for 2 weeks in the early development of the leaves at the beginning of GS2.

There were no significant effects of WL and soil Fr or their interactions with time on the photosynthesis variables (*A*_max_, *g*_s_, *E* and WUE) of leaves (see Table S2 available as Supplementary data at *Tree Physiology* Online). At the beginning of the second growing season, *A*_max_ of both higher and lower leaves was in the range of 25–28 μmol m^−2^ s^−1^. This increased to 33–38 μmolm^−2^ s^−1^ during the leaf expansion and decreased to approximately 20 μmolm^−2^ s^−1^ at the end of the long-day phase of the second growing season. Seasonal trends for *g*_s_, *E* and WUE were similar to those of *A*_max_.

Fr or WL did not affect the starch content of leaves. The interaction of soil Fr and time was significant, the starch content being higher in Fr than in NoFr at the end of the growing season (Day 232) before the start of the short-day treatment (Figure 4C, Table 2).

**Figure 5.**
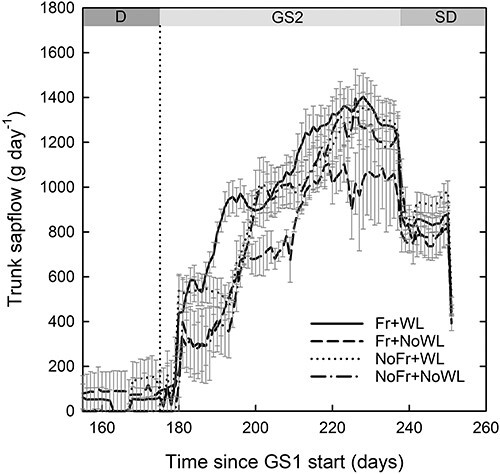
Daily mean trunk sap flow of silver birch saplings during the post-treatment dormancy period (D) and growing season (GS2), including the short-day (SD) period, in the experiment, with factorial combinations of WL and soil Fr during dormancy (D) (see Table 1). The vertical dotted line indicates the start of GS2. Time indicates running day from the beginning of the experiment. Bars indicate standard errors (*N* = 4).

For the stem sap flow, the interactions of soil Fr and WL with time were significant at the beginning of the growing season between Days 175 and 200 (Figure 5, Table 2). Between Days 185 and 195, the sap flow was highest in Fr + WL, but it did not significantly differ from the other treatments. The sap flow was lower in Fr + NoWL than in Fr + WL between Days 205 and 208, but in the latter part of the growing season, the differences between the treatments disappeared.

### Aboveground growth

**Figure 6.**
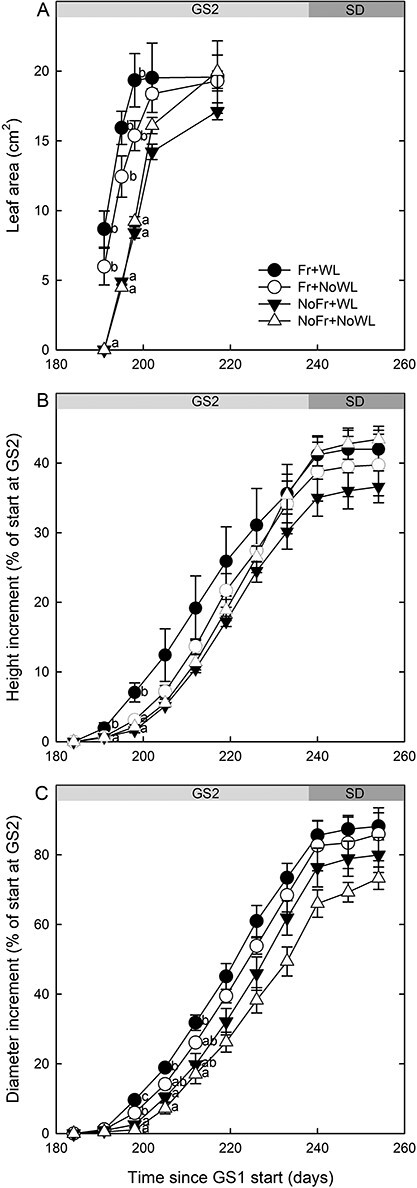
Growth of leaf area (pooled for upper and lower leaf) (A), elongation of shoot (B) and growth of trunk diameter (C) of silver birch saplings during the post-treatment growing season GS2, including the short-day period (SD), in the experiment, with factorial combinations of WL and soil Fr during dormancy. Shoot elongation and diameter growth are given as a percentage of the initial length and diameter in GS2. Significant differences by sampling times are indicated by different letters (*P* < 0.05). Time indicates running day from the beginning of the experiment. Bars indicate standard errors (*N* = 4).

Leaf unfolding and expansion took place earlier when the soil was exposed to Fr than not Fr (Figure 6A, Table 2). Relative shoot height growth was not affected by soil Fr or WL, but the interactions of Fr × WL and Fr × Time were significant; the relative growth being slightly higher in Fr + WL than in the other treatments at the beginning of the second growing season (see Days 198–226) (Figure 6B, Table 2). The relative growth of the stem diameter increased by soil Fr as compared with not Fr, and it was higher with than without WL (Figure 6C, Table 2). The interaction of soil Fr with time was significant, and the diameter growth started earlier in the treatment with than without soil Fr. The average final height and stem diameter (±SE) of all saplings were 150 ± 4 cm and 14.0 ± 0.3 mm respectively, and there were no significant differences between the treatments.

### Fine root production, mortality and longevity

**Figure 7.**
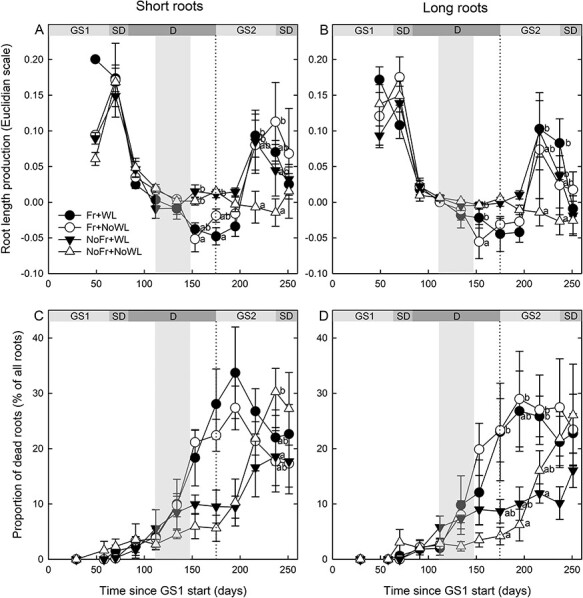
Short and long root production (A, B) and mortality (C, D) of silver birch saplings in the experiment, with factorial combinations of WL and soil Fr during dormancy. GS1 and GS2 refer to the growing seasons, including the short-day (SD) periods, and D to dormancy. The treatment period is indicated by the shaded column. The production is calculated as the difference of growth in the Euclidian normalized scale between two consequent imaging times. Significant differences by sampling times are indicated by different letters (*P* < 0.05). The vertical dotted line indicates the start of GS2. Time indicates running day from the beginning of the experiment. Bars indicate standard errors (*N* = 4).

There were no significant main effects of soil Fr or WL on short and long root production in the second growing season (Figure 7A and B, Table 2). The interaction of soil Fr with time was significant both for short and long roots (Table 2), production being lower by Fr than not Fr at the beginning of the second growing season. Production was minor or even turned negative at the beginning of the growing season, but it increased in the middle of the growing season in all treatments except NoFr + NoWL.

Soil Fr increased fine root mortality (Figure 7C and D). The interaction of soil Fr with time was significant for both short and long roots (Table 2). The first indications were observed during dormancy, and some differences were observed when the second growing season had started (*P* = 0.098 and *P* = 0.087 for short roots and *P* = 0.10 and *P* = 0.077 for long roots on Days 175 and 195, respectively). Waterlogging or its interaction with time had no significant effects on root mortality.

**Figure 8.**
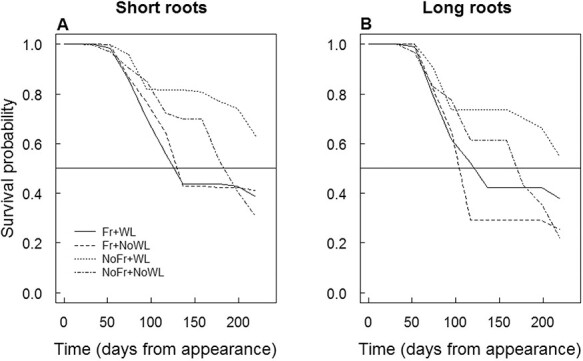
The survival probability of short (A) and long (B) roots of silver birch saplings in the experiment, with factorial combinations of WL and soil Fr during dormancy. The horizontal line at the survival probability level of 0.5 gives a measure of the median root longevity.

According to the survival analysis of the pooled data of all roots, soil Fr decreased the median longevity of short and long roots (Figure 8). For short roots, it was approximately 130 days in both soil Fr treatments, 190 days in NoFr + NoWL but longer than 200 days for NoFr + WL. In long roots, the respective values were between 105 and 125 days for the soil Fr treatments, 170 days for NoFr + NoWL and >200 days for NoFr + WL.

### Electrical impedance of roots and root hydraulic conductance

At the final harvest, the impedance loss factor of the roots was lower without than with soil Fr (Tables 2 and 3). A partly similar effect was observed in the stem above the root collar, but there was no effect in the soil. Root hydraulic conductance was higher if the roots were exposed to soil Fr than without soil Fr (Table 2) and more specifically, higher in Fr + WL than in NoFr + NoWL (*P* < 0.01) (Table 3).

**Table 3:** The mean (±SD) impedance loss factor (50 kHz) of roots, soil and stem and of reverse-flow root hydraulic conductance (*K*_r_) of silver birch seedlings in the experiment, with factorial combinations of soil Fr and WL during dormancy, and measured at the final harvest at the end of growing season GS2. Significant differences (*P* < 0.05) between the treatments are indicated with different letters (*N* = 4)

Treatment	Loss factor			*K*_r_, gs^−1^ MPa^−1^
	Root	Soil	Stem	
Fr + WL	−0.29 ± 0.06ab	−0.06 ± 0.02	−0.20 ± 0.02ab	0.220 ± 0.056a
Fr + NoWL	−0.25 ± 0.05b	−0.06 ± 0.02	−0.16 ± 0.04b	0.170 ± 0.066ab
NoFr + WL	−0.41 ± 0.05a	−0.10 ± 0.03	−0.26 ± 0.03a	0.118 ± 0.019ab
NoFr + NoWL	−0.38 ± 0.09a	−0.07 ± 0.01	−0.24 ± 0.03a	0.081 ± 0.025b

### Morphology of leaves

At the end of the long-day period of the second growing season, there were fewer non-glandular trichomes on the upper side of the short shoot leaves in the treatment without than with the soil Fr (Tables 2 and 4). Otherwise, there were no effects of soil Fr and WL on the glandular and non-glandular trichomes of leaves of either short or long shoots. There were more non-glandular trichomes on the upper than the lower side of the leaves in the short shoots in all treatments and in the leaves of the long shoots of Fr + NoWL (Table 4). The density of glandular trichomes was higher on the lower than upper sides of the short shoot leaves in NoFr + WL and in the long shoot leaves in Fr + WL, Fr + NoWL and NoFr + NoWL (Table 4). The stomatal density changed with time, but there were no effects of the treatments (data not shown). In short shoots, the density was the highest at the first sampling at the beginning of the second growing season (from 90 to 98 stomata mm^-2^ among the treatments) and the lowest at the second sampling before the start of the short-day period of the growing season (from 71 to 79 stomata mm^-2^ among the treatments). The density in long shoot leaves ranged from 127 to 143 stomata mm^-2^.

**Table 4:** The mean density (±SD) of glandular and non-glandular trichomes (number of trichomes per mm^2^ of leaf area) on the upper and lower side of the leaves of short and long shoots of silver birch in the experiment, with factorial combinations soil Fr and WL) during dormancy, as counted at the end of the long-day phase of the post-treatment growing season (*n* = 4, except *n* = 3 for non-glandular trichomes on the upper side of long shoot leaves NoFr + NoWL and *n* = 2 for non-glandular trichomes on the lower side of long shoot leaves of NoFr + NoWL). The different lower-case letters indicate the significant differences between the treatments (*P* < 0.05); the different capital letters indicate between the sides of the leaves by treatments (up vs down, paired samples *t*-test) (*P* < 0.05). No letter indicates no difference.

Trichome type	Treatment	Short shoot leaf		Long shoot leaf	
		Upper side	Lower side	Upper side	Lower side
Glandular, no. mm^−2^	Fr + WL	2.02 ± 0.37	2.13 ± 0.41	3.24 ± 0.86B	3.86 ± 0.97A
	Fr + NoWL	2.08 ± 0.48	2.30 ± 0.36	2.73 ± 0.55B	3.83 ± 0.59A
	NoFr + WL	2.73 ± 1.11B	3.01 ± 1.07A	3.47 ± 0.50	4.01 ± 0.16
	NoFr + NoWL	2.31 ± 0.34	2.53 ± 0.38	3.28 ± 0.39B	4.15 ± 0.81A
Non-glandular, no. mm^−2^	Fr + WL	2.72 ± 1.03aA	0.10 ± 0.14B	0.19 ± 0.21	0.70 ± 0.48
	Fr + NoWL	2.29 ± 0.59aA	0.03 ± 0.06B	0.03 ± 0.06B	0.69 ± 0.15A
	NoFr + WL	1.42 ± 0.32bA	0.09 ± 0.17B	0.62 ± 0.50	0.76 ± 0.19
	NoFr + NoWL	1.36 ± 0.30bA	0 ± 0B	0.21 ± 0.04	0.88 ± 0.46

### Biomass and area of leaves, biomass of roots

**Figure 9.**
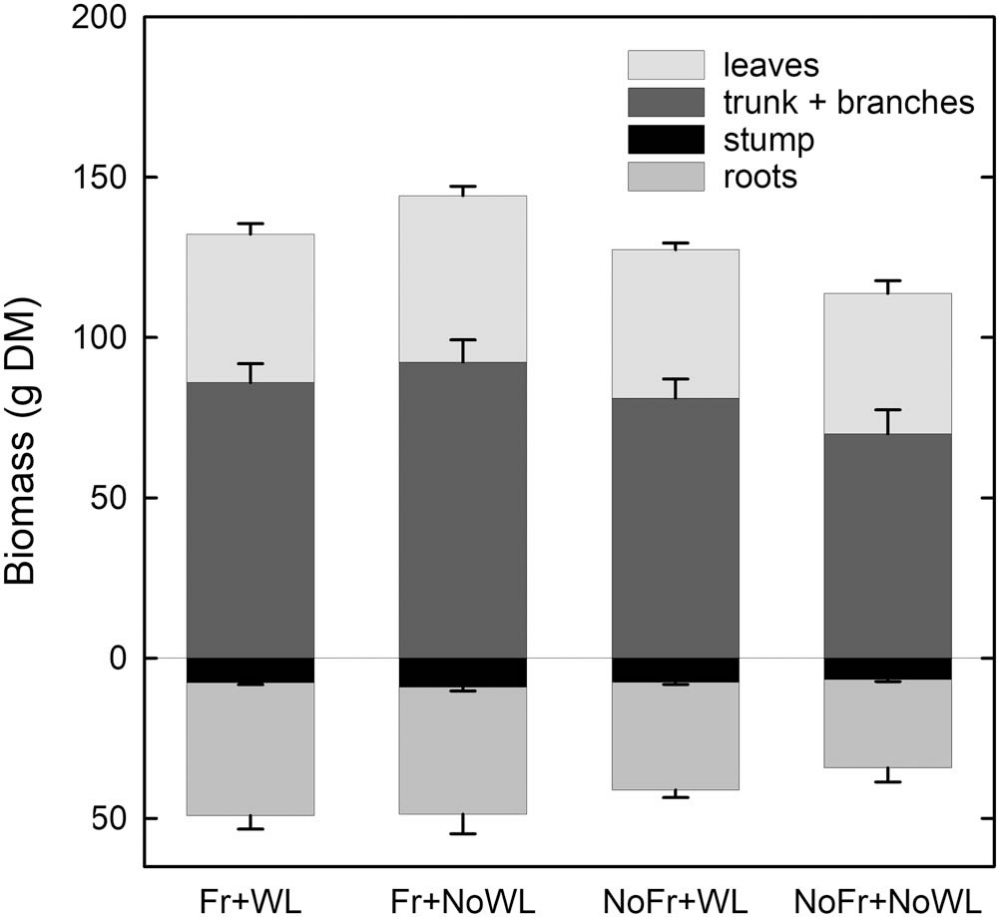
Dry biomass of different cohorts of silver birch seedlings in the experiment with WL and soil Fr during dormancy as assessed at the final harvest at the end of the post-treatment growing season. The data of short and long shoots are pooled. Bars indicate the standard errors of the mean (*N* = 4).

At the final harvest, the effect of soil Fr and WL or their interaction on the total dry mass was not significant (Figure 9, Table 2). There were no significant differences between the treatments in the dry mass of leaves, stems (including branches) (Figure 9) and in the surface area of leaves, or in the total length of stems and branches, in either short or long shoots (results not shown). The leaf dry mass of long shoots ranged from 36 g per sapling (NoFr + NoWL) to 43 g per sapling (Fr + NoWL), whereas the leaf dry mass of short shoots was on average 11 g per sapling. The number of leaves varied between 460 and 690 leaves per sapling, without significant differences between the treatments.

## Discussion

### Shoot and root responses

Leaf unfolding and expansion took place earlier if exposed to soil Fr than not Fr in both WL treatments. This accords with our previous study for the start of shoot elongation of Scots pine seedlings in similar WL and soil Fr treatments as in this study (Roitto et al. 2019). Although the air conditions were similar in all treatments, it is possible that the chilling effect of frozen soil was mediated from the roots to the buds by some unknown mechanisms. This enhanced the break of rest in buds and recovered their growth competence, leading to decrease of time to bud burst and growth initiation in the forcing conditions. Another explanation is that higher soil temperatures in the treatments without Fr increased the respiration and consumption of stored carbohydrates during dormancy (Ögren et al. 1997). During growth onset, the shortage of carbohydrates would therefore have delayed the start of shoot elongation due to the sink effect of roots for stored photosynthates. The early start of leaf expansion in the treatment with soil Fr was also reflected in the growth rhythm of shoot elongation and diameter growth, but the differences in growth had disappeared by the end of the recovery growing season. Therefore, the loss of fine roots (see below) did not cause measurable losses in aboveground functions. The situation might have been different in conditions of water or nutrient deficits, which is a topic for further studies.

The dark-acclimated chlorophyll fluorescence (*F*_v_/*F*_m_) and CCI of leaves increased earlier in the treatment with than without soil Fr. The similar responses of these variables and leaf expansion suggest that the dynamics of *F*_v_/*F*_m_ and the CCI were due to the different ages of the leaves. In contrast, gas exchange did not show treatment effects. This is probably because the gas exchange measurements started later than *F*_v_/*F*_m_ and the CCI, i.e., just after the leaves were large enough to cover the chamber aperture, and at that time the difference in leaf age was no longer a significant factor. In addition, the result on gas exchange agrees with the result of no difference in the stomatal densities between treatments. Waterlogging did not affect any of these variables. In the previous study with silver birch seedlings, the CCI and *F*_v_/*F*_m_ increased less in the post-treatment growing season if the WL took place at the end of dormancy than at the beginning of the growing season (Wang et al. 2017). In contrast, in downy birch (*B. pubescens*), no such effect was observed, suggesting different survival strategies and WL tolerance between these two birch species. Although we did not observe differences in *A*_max_ among the treatments in silver birch, the starch content of leaves was temporarily higher in Fr than in NoFr saplings in the latter part of the growing season, before the cessation of shoot elongation. This may be connected with the change in resource allocation between roots (mortality, repair of damage) and shoots (decrease in growth). In the previous study with similar experimental set-up as here, *F*_v_/*F*_m_ of Scots pine needles was lower in Fr than in NoFr during dormancy, the difference disappeared when the growing season started but there were no effects of WL (Roitto et al. 2019). As opposed to the results here, the WL of Scots pine saplings in the middle of the growing season decreased *A*_max_ and *F*_v_/*F*_m_ after the 3 weeks of the treatment (Repo et al. 2016).

In the post-treatment period at the end of dormancy and at the beginning of the growing season, the length production of short and long roots was lower (even negative) if exposed to soil Fr than without Fr, and in the middle of the growing season lower in waterlogged than not waterlogged treatment. Negative production coincides with increased mortality in the treatment with soil Fr as compared with without soil Fr. The shorter lifetime of the fine roots in the treatment with soil Fr than not Fr is also in agreement with the results of fine root mortality and production. Furthermore, the increased hydraulic conductance and impedance loss factor of the frost-exposed roots indicate that there were still after-effects of the soil Fr treatment (cf. Di et al. 2019, Korhonen et al. 2019), even though the biomass had recovered.

In accordance with this study, the short root mortality of Scots pine saplings increased, and their longevity decreased in frozen soil, but a soil WL treatment did not have similar effects (Roitto et al. 2019). In another controlled-environment experiment, repeated soil freeze–thaw cycles (using simulated rain on frozen soil) were harmful for fine roots of Scots pine saplings during dormancy, inducing xeromorphic features in the needles that developed after the treatments (Sutinen et al. 2014). In a field experiment with snow removal, soil Fr increased fine root mortality in a northern hardwood forest in New Hampshire (USA), dominated by American beech (*Fagus grandifolia* Ehrh.), sugar maple (*Acer saccharum* Marsh.) and yellow birch (*Betula alleghaniensis* Brit.) (Groffman et al. 2001, Tierney et al. 2001, 2003), and in a Norway spruce stand in southeast Germany (Gaul et al. 2008). It was suggested that the increased fine root mortality was due to mechanical damage by soil movement due to Fr and thawing, and low soil temperatures were not therefore the sole cause of root damage (Tierney et al. 2003, Sutinen et al. 2014). Yet increased soil Fr in a 50-year-old stand of Norway spruce had no clear adverse effects on fine root longevity or mortality, even though the seasonal dynamics of root growth were altered by snow manipulations (Repo et al. 2014*b*). This indicates that if roots are not severely damaged, they may recover (Coursolle et al. 2002). As found in this and the previous studies, enhanced fine root production in some later phases of the growing season may compensate for earlier growth losses (Tierney et al. 2003, Roitto et al. 2019). A possible explanation is a nutrient pulse due to soil Fr from damaged roots and microbes that led to increased root growth with delay (Cleavitt et al. 2008, Sanders-DeMott et al. 2018). No costs for the compensatory growth of roots were found in the aboveground organs, but the shoot and stem diameter growth were even higher with than without soil Fr. However, possible long-term effects in later growing seasons, with annually repeating Fr and thawing, were not topic of this study.

Stem sap flow was not affected by WL and soil Fr alone, but their interaction with time was significant at the beginning of the growing season, the sap flow being higher in Fr + WL than in the other treatments. In all treatments, the stepwise increase of sap flow was probably connected with the phenological events in shoots and roots, i.e., the timing of the growth of short and long shoots, and with the change in leaf area accordingly (Smith et al. 1996, Santiago et al. 2000). After the first rapid increase of sap flow at the beginning of the growing season, there was a short steady flow period. No growth or minor growth of fine roots was observed during this period, and the expansion of the first leaves took place a little later. Surprisingly, no effects of gradually increasing fine root mortality in the treatments with soil Fr were seen in the stem sap flow, which may be explained by the abundant availability of water in all treatments. Sap flow reached its maximum at the end of the long-day period of the growing season, when shoot elongation was ending, and the transpiring leaf area attained its maximum. At that time, fine root production also reached its maximum, except in NoFr + NoWL, where root production was low throughout the growing season. The onset of the short-day period at the end the growing season followed by the lowering of soil and air temperatures appeared as a rapid decrease in sap flow, coinciding with the decrease in the growth rates of shoots and roots.

At the end of the second growing season, the non-glandular trichome (hairs) density on the upper surface of the leaves of short shoots increased after soil Fr as compared with the treatment without Fr. This indicates inducible legacy effects of soil Fr during dormancy on morphology in leaves. Non-glandular trichomes are typically more abundant in pubescent than silver birch, which is less adapted to soils with excess water (Wang et al. 2016). However, it seems that silver birch may also respond to soil Fr by increasing the hair density of the leaves. Non-glandular trichomes that do not excrete phytochemicals are considered to enhance plant protection against adverse factors merely mechanically, whereas glandular trichomes are important mainly in chemical defense (Oksanen 2018). Thitz et al. (2017) suggested that glandular trichomes, as well as non-glandular trichomes, may affect plant acclimation to different air temperature and soil moisture conditions through possible effects on the transpiration and temperature control of the leaves. In our study, during dormancy, WL and soil Fr did not affect glandular trichomes, opposite to a previous study, where WL at the beginning of the growing season increased them in silver birch (Wang et al. 2016).

### Soil gas concentrations

The soil O_2_ content responded immediately to the changes in the water table, indicating a physical process for its replacement. The concentrations of the other gases, i.e., CO_2_, CH_4_ and N_2_O, changed more slowly only in the post-treatment period during dormancy and/or at the beginning of the growing season, indicating biochemical processes in their production. The small differences in soil water content after the treatments seemed not to affect soil gas concentrations and likely had no significant effects on the root and shoot response, but the differences in the soil gas concentrations between treatments were mostly observed whether soil was frozen or not.

The most distinctive changes and differences between the treatments in soil gas concentrations were observed in the upper soil level. Freezing caused an accumulation of CO_2_ in the soil and appeared as an elevated concentration by thawing in accordance with the previous studies concerning CO_2_ emissions (van Bochove et al. 2001, Kurganova et al. 2007, Maljanen et al. 2010). There were two phases in the increase of CO_2_ concentration after soil thawing. The first short-term peak was probably due to a physical process in which trapped CO_2_ during dormancy was released by soil thawing (Kurganova et al. 2007). This peak was followed by a slow increase during the growing season in all treatments, which can be explained by an increase of root and microbial activity and root respiration due to the temperature increase. Since the microbial biomass and activity/mortality (Groffman et al. 2001 and other references cited there), as well as fine root mortality, have been found to be sensitive to freeze–thaw cycles, a long-term root respiration increase and a CO_2_ concentration increase would therefore have been expected in the soil Fr treatments too. However, this effect was not observed, apart from a slightly earlier increase only in Fr + WL treatment. More surviving roots in the treatments without soil Fr probably respired more, while there may have been a higher decomposer activity of freeze-damaged roots and microbes after the treatments with soil Fr. Further studies separating the root and microbial respiration after soil frost could show if this is the case.

The concentration of CH_4_ during the post-treatment period was lower in soils exposed to Fr than without Fr, but there was no difference between the WL treatments. This indicates that there was CH_4_ accumulation in unfrozen soil during dormancy, irrespective of WL. Although there are few studies concerning the CH_4_ fluxes in varying winter conditions, fluxes have been shown to be sensitive to snowpack properties (Borken et al. 2006). In a temperate forest soil, snow cover significantly reduced the influx of CH_4_ but increased after snow removal, as long as the soil was not frozen (Borken et al. 2006), but decreased with soil Fr (Groffman et al. 2006). Because there were no differences between the WL treatments, we may conclude that no additional CH_4_ production took place by anaerobic microorganisms.

In contrast with the seasonal changes in the concentration of CO_2_ and CH_4_, no seasonal pattern was found in N_2_O, which agrees with a previous study concerning fluxes (Groffman et al. 2006). It has been noted that the N_2_O fluxes are heterogeneous, and there is wide variation in the estimates of winter fluxes in different ecosystems (Groffman et al. 2000). In our study, a strong short-term N_2_O concentration peak was found at the beginning of the growing season only in Fr + WL treatment. Then, the concentration remained slightly elevated until the end of the growing season, which may also have led to elevated N_2_O emissions. Due to the frequent sampling interval (twice per week), the peak was probably close to the potential maximum. The peak occurred after the first CO_2_ burst and at the time when CO_2_ concentration had started to increase and CH_4_ concentration had reached the low stable level. In accordance with our results for concentration, increased N_2_O effluxes have been found by freeze–thaw events during the winter and during the transition from winter to spring too (Brumme et al. 1999, Groffman et al. 2000, 2006, Maljanen et al. 2010). There are several possible explanations for the increased N_2_O emissions by soil Fr, including a reduced plant uptake of nitrogen due to increased root mortality, as was found in the present study, leading to its transformation into N_2_O, increased Fr-induced microbial mortality and disruption of soil aggregates, stimulating denitrification (Groffman et al. 1993, 2006).

## Conclusions

A whole-tree approach was used to study the reactions of silver birch saplings to WL and soil Fr during dormancy and the changes in the soil gas concentrations by the treatments. In our experiment in controlled conditions, more consistent changes in roots and shoots were observed by soil Fr than by WL. The activation of physiological processes and growth in the aboveground organs took place earlier if the roots were exposed to soil Fr than without Fr. Fine root mortality increased by soil Fr at the beginning of the post-treatment growing season, followed by compensatory growth of fine roots in the later part of the growing season. However, the fine root function did not completely recover by the end of the growing season, when another winter would follow in field conditions. As the frost effects on roots are not well known even in the present climatic conditions, also basic studies into repeated Fr are required. In contrast, no effects or only temporary effects of WL were observed, which corroborates earlier findings on stronger effects of WL in the growing season under than dormancy conditions. The soil gas concentrations support the observations of increased root damage by soil Fr. The changes in gas concentrations most probably are a result of increased decomposer activity and nutrient leaching into soil due to increased root damage. According to the climate scenarios for winter precipitation in the boreal zone, soil conditions are in transition and will change within the coming decades. Whether the precipitation falls as snow or rain, winter floods may become more common, and soil Fr may increase or decrease. According to the present study, the increasing variability in soil Fr and thawing deserves further attention, while winter-time WL effects on boreal trees are less severe than those of growing season WL.

## Supplementary Material

Supplement_Table_S2_rev_clean_tpab002
