# Supplementary material for: Dynamics of above- and belowground responses of silver birch saplings and soil gases to soil freezing and waterlogging during dormancy
Source: Tree Physiol. 2021 Jan 13;41(7):1143–60. doi: 10.1093/treephys/tpab002 (PMC8271213; doi:10.1093/treephys/tpab002)
Supplement: Supplement_Table_S2_rev_clean_tpab002 [file supplement_table_s2_rev_clean_tpab002.docx]

**Table S2.** Summary of the statistics by linear mixed model (degrees of freedom, F-value, P-value) on the effects of soil freezing (Fr) and waterlogging (WL) during dormancy on silver birch saplings during the follow-up growing season The density of non-glandular and glandular trichomes was analyzed separately for upper (‘up’) and lower (‘down’) surfaces of the leaves of ‘short’ and ‘long’ shoots. ‘Short’ and ‘long’ roots refer to the 1^st^ and higher than the 1^st^ order roots respectively. The results of two-way Anova only is presented for the data at the final harvest. For the symbols and abbreviations see the table ‘List of symbols and abbreviations’.

| *Shoot physiology and growth* | df1; df2; F-value; [P-value] | | | | |
| --- | --- | --- | --- | --- | --- |
|  | Fr | WL | Fr x WL | Fr x Time | WL x Time |
| F_v_/F_m_ (short) | 1; 4.6; 7.3; [**0.046]** | 1; 22.3; 0.02; [0.89] | 1; 24.8; 1.71; [0.20] | 13; 29.7; 7.0; [**<0.001**] | 15; 21.0; 1.92; [0.08] |
| F_v_/F_m_ (long) | 1; 15.2; 0.16; [0.70] | 1; 15.2; 0.82; [0.38] | 1; 15.2; 1.33; [0.27] | 4; 25.4; 0.99; [0.43] | 4; 25.4; 3.66; [**0.02**] |
| CCI | 1; 36.1; 58.3; [**<0.001]** | 1; 36.1; 1.6; [0.22] | 1; 36.1; 0.91; [0.35] | 19; 213.5; 7.6; [**<0.001**] | 19; 213.5; 2.43; [**<0.01**] |
| A_max_ | 1; 2.4; 0.07; [0.82] | 1; 17.8; 2.0; [0.17] | 1; 17.8; 0.3; [0.59] | 3; 19.7; 1.6; [0.21] | 3; 19.7; 0.34; [0.80] |
| g_s_ | 1; 2.1; 0.3; [0.65] | 1; 15.1; 0.9; [0.37] | 1; 15.1; 2.4; [0.14] | 3; 22.8; 0.69; [0.57] | 3; 22.8; 1.0; [0.42] |
| E | 1; 2.7; 0.6; [0.49] | 1; 21.1; 1.5; [0.23] | 1; 21.1; 0.5; [0.48] | 3; 18.3; 0.4; [0.75] | 3; 18.3; 0.4; [0.78] |
| WUE | 1; 2.4; 0.05; [0.84] | 1; 17.2; 2.0; [0.18] | 1; 17.2; 0.3; [0.60] | 3; 19.7; 1.7; [0.19] | 3; 19.7; 0.3; [0.79] |
| Starch content | 1; 16.0; 3.1; [0.10] | 1; 16.0; 0.63; [0.44] | 1; 16.0; 0.25; [0.62] | 4; 26.8; 3.0; [**0.04**] | 4; 26.8; 0.72; [0.58] |
| Sap flow (P1) | 1; 148; 0.37; [0.54] | 1; 141; 3.6; [0.06] | 1; 141; 0.20; [0.66] | 25; 134; 3.1; [**<0.001**] | 25; 134; 4.1; [**<0.001**] |
| Sap flow (P2) | 1; 0.12; 0.13; [0.90] | 1; 1596; 1.9; [0.17] | 1; 1596; 2.0; [0.16] | 19; 232; 3.8; [**<0.001**] | 19; 232; 0.87; [0.62] |
| Sap flow (P3) | 1; 5.1; 0.83; [0.40] | 1; 11.6; 2.6; [0.14] | 1; 11.6; 1.5; [0.25] | 16; 69.6; 0.64; [0.84] | 16; 69.6; 1.1; [0.39] |
| Sap flow (P4) | 1; 5.0; 0.2; [0.66] | 1; 0.37; 2.3; [0.57] | 1; 0.37; 0.11; [0.85] | 12; 2.9; 0.93; [0.61] | 12; 2.9; 0.38; [0.90] |
| Shoot elongation | 1; 2.0; 3.58; [0.20] | 1; 17.0; 0.75; [0.40] | 1; 17.0; 7.0; [**0.02**] | 10; 83.4; 5.0; [**<0.001**] | 10; 83.4; 1.8; [0.08] |
| Stem diameter growth | 1; 18.7; 27.2; [**<0.001]** | 1; 18.7; 6.0; [**0.03**] | 1; 18.7; 0.09; [0.77] | 10; 106.5; 7.3; [**<0.001**] | 10; 106.5; 1.57; [0.13] |
| Leaf expansion | 1; 2.9; 89.2; [**0.003]** | 1; 11.2; 0.9; [0.38] | 1; 11.2; 3.2; [0.10] | 4; 29.6; 62.8; [**<0.001**] | 4; 29.6; 2.8; [**0.046**] |
| DM (leaves) | 1; 1.67; [0.220] | 1; 0.22; [0.646] | 1; 1.67; [0.226] | n.d. | n.d. |
| DM (stem & branches) | 1; 4.26; [0.061] | 1; 0.08; [0.786] | 1; 2.08; [0.175] | n.d. | n.d. |
| *Leaf morphology* |  |  |  |  |  |
| Non-gland. (short, up) | 1; 12.3; [**0.004]** | 1; 0.58; [0.46] | 1; 0.35; [0.57] | n.d. | n.d. |
| Non-gland. (short,down) | 1; 0.137; [0.72] | 1; 1.68; [0.22] | 1; 0.03; [0.86] | n.d. | n.d. |
| Non-gland. (long, up) | 1; 4.22; [0.07] | 1; 3.65; [0.08] | 1; 0.69; [0.43] | n.d. | n.d. |
| Non-gland. (long, down) | 1; 0.50; [0.50] | 1; 0.09; [0.78] | 1; 0.13; [0.73] | n.d. | n.d. |
| Gland. (short, up) | 1; 2.04; [0.18] | 1; 0.29; [0.60] | 1; 0.55; [0.48] | n.d. | n.d. |
| Gland. (short, down) | 1; 3.15; [0.10] | 1; 0.23; [0.64] | 1; 1.05; [0.33] | n.d. | n.d. |
| Gland. (long, up) | 1; 1.70; [0.22] | 1; 1.35; [0.27] | 1; 0.31; [0.59] | n.d. | n.d. |
| Gland. (long, down) | 1; 0.45; [0.52] | 1; 0.02; [0.88] | 1; 0.06; [0.81] | n.d. | n.d. |
| Stomata (short) | 1; 11.9; 0.01 [0.94] | 1; 11.9; 0.25; [0.63] | 1; 11.9; 4.16; [0.06] | 2; 18.1; 0.58; [0.57] | 2; 18.1; 0.35; [0.71] |
| Stomata (long) | 1; 1.55; 0.33; [0.64] | 1; 10.5; 0.01; [0.94] | 1; 10.5; 0.51; [0.49] | n.d. | n.d. |
| *Roots* |  |  |  |  |  |
| Production (short) | 1; 2.5; 0.02; [0.91] | 1; 11.2; 0.4; [0.53] | 1; 11.2; 4.6; [0.055] | 4; 18.2; 8.1; [**0.001**] | 4; 18.2; 2.4; [0.08] |
| Production (long) | 1; 22.9; 0.4; [0.52] | 1; 22.9; 2.2; [0.15] | 1; 22.9; 0.02; [0.89] | 10; 24.6; 3.5; [**0.005**] | 10; 24.6; 2.4; [**0.04**] |
| Mortality (short) | 1; 2.1; 0.5; [0.54] | 1; 13.7; 0.05; [0.83] | 1; 13.7; 1.4; [0.26] | 9; 38.9; 8.1; [**<0.001**] | 9; 38.9; 0.9; [0.55] |
| Mortality (long) | 1; 2.0; 0.9; [0.43] | 1; 16.7; 0.1; [0.76] | 1; 16.7; 0.2; [0.66] | 9; 42.6; 4.3; [**<0.001**] | 9; 42.6; 1.2; [0.32] |
| Loss factor (EIS) | 1; 16.1; [**0.002**] | 1; 1.01; [0.33] | 1; 0.06; [0.82] | n.d. | n.d. |
| Hydr. conductance | 1; 17.0; [**0.001**] | 1; 3.58; [0.08] | 1; 0.07; [0.80] | n.d. | n.d. |
| DM (stump) | 1; 2.40; [0.147] | 1; 0.08; [0.777] | 1; 1.94; [0.189] | n.d. | n.d. |
| DM (roots) | 1; 4.41; [0.057] | 1; 0.58; [0.460] | 1; 0.20; [0.663] | n.d. | n.d. |
| *Soil gases* |  |  |  |  |  |
| CO_2_ (top) | 1; 3.6; 0.9; [0.41] | 1; 19.0; 8.7; [**0.008**] | 1; 19.0; 0.40; [0.54] | 20; 48.5; 2.3; [**0.01**] | 20; 48.5; 3.6; [**<0.001**] |
| CO_2_ (bottom) | 1; 25.8; 3.1; [0.09] | 1; 26.5; 2.2; [0.15] | 1; 26.5; 0.16; [0.69] | 20; 50.7; 1.8; [0.054] | 20; 50.7; 1.1; [0.38] |
| CH_4_ (top) | 1; 5.6; 8.7; [**0.028]** | 1; 36.3; 0.001; [1.0] | 1; 36.3; 1.0; [**<0.001**] | 20; 38.7; 3.1; [**0.001**] | 20; 38.7; 0.9; [0.59] |
| CH_4_ (bottom) | 1; 12.4; 7.4; [**0.018]** | 1; 45.4; 0.14; [0.72] | 1; 45.4; 1.67; [0.20] | 20; 38.4; 2.5; [**0.007**] | 20; 38.4; 1.8; [0.056] |
| N_2_O (top) | 1; 3.8; 7.9; [0.052] | 1; 20.6; 34.7; [**<0.001**] | 1; 20.6; 0.9; [0.35] | 20; 38.8;9.4; [**<0.001**] | 20; 38.8; 8.3; [**<0.001**] |
| N_2_O (bottom) | 1; 11.7; 0.3; [0.59] | 1; 38.1; 5.9; [**0.02**] | 1; 38.1; 1.0; [0.32] | 20; 33.1; 2.4; [**0.012**] | 20; 33.0; 2.2; [**0.024**] |
